# From evidence to decisions: building knowledge translation for pandemic preparedness and response

**DOI:** 10.1186/s12919-026-00410-3

**Published:** 2026-11-10

**Authors:** Sara Bennett, Verena Struckmann, Barbara Tornimbene, Firmaye Bogale, Shelly-Ann Hunte, Tanja Kuchenmüller, Samuel Mbugua

**Affiliations:** 1 Department of International Health, Johns Hopkins Bloomberg School of Public Health, Baltimore, MD USA; 2 Department of Health Care Management, Technical University of Berlin, Berlin, Germany; 3 CPC Analytics, Berlin, Germany; 4 Knowledge Translation Directorate, Ethiopian Public Health Institute, Addis Ababa, Ethiopia; 5 Faculty of Medical Sciences, Caribbean Centre for Health Systems Research and Development, The University of the West Indies, St. Augustine, Trinidad and Tobago, Kingston, Jamaica; 6 Science for Health Department, Science Division, World Health Organization, Geneva, Switzerland; 7 United States International University-Africa, Nairobi, Kenya

**Keywords:** Knowledge translation, Evidence-informed decision-making, Institutionalisation, Pandemic preparedness, Epidemic intelligence, Low- and middle-income countries

## Abstract

The COVID-19 pandemic showed that moving evidence quickly into decisions matters as much as strong surveillance, especially in low- and middle-income countries. During the fifteenth session of the WHO Pandemic and Epidemic Intelligence Innovation Forum, held on 24 February 2026, speakers from the World Health Organization, the Caribbean Centre for Health Systems Research and Development in Trinidad and Tobago, the United States International University-Africa in Kenya and the Ethiopian Public Health Institute discussed how evidence-to-decision processes are being built and how they perform during health emergencies. The Ethiopian Public Health Institute reported that a dedicated knowledge translation team within the emergency management system reduced the time between a decision maker’s question and an evidence response from days or months to hours, drawing on a mandate and relationships established long before the pandemic. In Trinidad and Tobago, a proposed rapid response service could not be established without government funding and engagement, and the momentum gained during COVID-19 faded as the urgency passed. Speakers described arrangements that help sustain evidence use, including the WHO Evidence-informed Policy Network and its six domains of institutionalisation, county-level taskforces, reverse research days and national research agendas. Political turnover, limited resources, unharmonised tools and declining trust in science were identified as the main obstacles. Artificial intelligence was seen as a way to accelerate evidence synthesis, but not to replace the relationships and dialogue at the core of knowledge translation. Moving forward, evidence systems need to be built in ordinary times so that they can support decisions when the next crisis comes.

## Introduction

During emergencies, health authorities often need to make critical decisions based on evidence that is incomplete and changing very rapidly. Epidemic intelligence can identify a threat, but its findings must be interpreted alongside research, service data and local experience before they can guide a response. This calls for regular exchange between those producing evidence and those responsible for decisions, as well as ways to summarise and discuss findings within a short time window.

The COVID-19 pandemic brought these needs into sharper focus, particularly in low- and middle-income settings where available evidence must often be adapted to local priorities and resources. Work on knowledge translation has sought to connect evidence generation with policy through dedicated teams, intermediary organisations, evidence briefs and dialogue with decision makers. Whether these efforts shape decisions depends on their relevance and timing, but also on trusted relationships, institutional mandates, staff capacity and political support.

On 24 February 2026, the WHO Hub for Pandemic and Epidemic Intelligence convened an Innovation Forum session [1] on how health systems can learn across settings and use evidence in preparedness and response. The session was moderated by the Johns Hopkins Bloomberg School of Public Health and the Technical University of Berlin, with contributions from the World Health Organization, the Caribbean Centre for Health Systems Research and Development (CCHSRD) at The University of the West Indies, the United States International University-Africa and county partners in Kenya, and the Ethiopian Public Health Institute. Together they explored how evidence use can become part of routine work and how these arrangements perform during emergencies. This report draws together their experiences and the subsequent discussion, with attention to the practical conditions that allow evidence to inform public health decisions. The examples reflect the practical experience reported by speakers rather than formally evaluated outcomes, and whether these approaches change decisions in practice remains an open question.

## The nature of evidence in public health decision-making

Public health authorities use evidence to set priorities, plan services and decide where to invest. In routine policy making there is usually time to gather evidence, consult stakeholders and compare options. Decision makers must weigh research and surveillance findings alongside feasibility, available resources, economic effects and public values. Pandemic and epidemic intelligence integrates surveillance data with contextual knowledge to assess health risks and inform public health decisions. Knowledge translation, which brings this evidence to decision makers in a form they can use, is therefore part of intelligence itself, not a separate step. The task is to identify which evidence is relevant, what remains uncertain and how it applies to a particular decision. Too little information can leave decision makers uncertain about the nature of a threat or the likely effects of a response. An abundance of findings can also make decisions harder when studies differ, evidence changes quickly, or the available material does not answer the question at hand.

Even when evidence is available, people may read the same evidence differently because they prioritise different aims: protecting health services and reducing deaths, for example, while limiting harm to livelihoods. Urgency also changes how evidence is applied. Authorities may need to introduce a measure while continuing to study its effects and taking into account considerations beyond evidence of effectiveness alone [2].

The path from research to policy passes through several stages, from setting research priorities and generating findings to making those findings available and considering them in policy. This takes place within a complex evidence-informed policy ecosystem that includes a large set of people and organisations [3]. Academia, surveillance teams, ministries, local authorities, civil society and the media have different roles in producing, interpreting and sharing evidence. Decision makers themselves range from technical staff to elected officials, who may ask different questions and weigh outcomes differently. Further, this occurs within the national context, its governance and structures, social and economic conditions, education and infrastructure, and it is influenced by a number of factors such as ideology and values, capacity to use the evidence, personal experience, interests and other external influences. This complexity becomes particularly critical in an emergency, when all these actors have to work together under time pressure.

## Bringing government and research together

Within the evidence-informed policy ecosystem, government and research are two central actors, and evidence is most likely to be used when they work together as equal partners. During an epidemic, decisions may have to be made with less certainty, greater disagreement and far less time to act, while the health system must already answer immediate questions about the threat and the response. An outbreak compresses the time available to answer a question and can change the question itself. Evidence briefs and rapid reviews can make findings easier to consider, but their value depends on the questions they answer and the relationships through which they are produced. Without them, useful work may be missed or reach the people who need it too late.

Both the Johns Hopkins Bloomberg School of Public Health and WHO emphasised the importance of this partnership, and WHO added that decision makers can also contribute practical and tacit knowledge. Involving them in co-creation supports a plurality of forms of evidence, alongside clear communication of the uncertainty that remains. Deliberative and participatory approaches allow evidence to be discussed with decision makers, rather than passively brought to their attention in the expectation that it will be used in a linear way.

A good example of how established relationships can get evidence to decision makers in time is the work done during COVID-19 by the Ethiopian Public Health Institute’s knowledge translation team, which mainly supports the Ministry of Health. A dedicated team was placed within the planning section of the country’s emergency management system. By engaging directly with decision makers on questions as they arose, the team reported that it reduced the interval between a request and an evidence response, which could otherwise take days or months, to hours. The Institute attributed much of what it achieved to a clear mandate and position in the health system as a knowledge broker, held for over 15 years, which gave it existing learning, structures and relationships to draw on when the emergency began.

Another example of how evidence is most likely to be used when government and research work together is the experience of Trinidad and Tobago, which shows how valuable such a link can be, and what happens when it is incomplete. In 2016, a conversation between the then Minister of Health and the Dean of the Faculty of Medical Sciences at The University of the West Indies brought a problem into focus: the university was producing research, but it did not consistently answer policy makers’ questions. A feasibility study and consultations followed, and CCHSRD was established in 2018 to connect research with policy needs. The centre assessed what ministries needed, then developed tailored training, an Evidence to Policy fellowship, evidence briefs and dialogue with decision makers. It also organised a reverse research day, an event that reverses the usual roles so that policy makers present their research needs to researchers [4]. At the event, held with the Ministry of Health in July 2022, policy makers from each strategic priority area, including communicable diseases, health systems and oral health, presented the questions they most needed answered for their policy agendas, while researchers from three local universities listened from the audience. Participants then worked in groups to establish whether the research already existed and policy makers were unaware of it, whether unpublished data existed, or whether the ministry could provide the data needed. Participants also committed to developing joint research proposals; at a one-year follow-up these had not materialised, but the results informed a national health research agenda.

## Why momentum fades

Despite these gains, partners saw interest in better evidence use fade as the urgency passed, or fail to take hold where government engagement was missing. In early 2020, CCHSRD sought to establish a rapid response service that could turn locally relevant evidence into concise briefs answering policy makers’ questions within 3 to 30 business days. The proposed rapid response service could not be established without government seed funding and engagement, despite the capacity of CCHSRD to prepare evidence briefs. In Ethiopia, the momentum built during COVID-19 was also not fully carried into routine work.

These limits raise a long-term question: how to embed evidence use in the structures, processes and culture of the health system, so that it does not depend on an emergency or on particular individuals. The relationships between those who produce evidence and those who use it, and the political conditions around them, could matter as much as the evidence itself. WHO emphasised the importance of personal relationships and trust between researchers and decision makers [5–7], especially during health emergencies, when decisions must be made quickly and direct contact is paramount to ensure evidence is timely and relevant. But these relationships need to be built into institutional structures and maintained to become part of the culture so they can endure political cycles. The aim is a joint ecosystem for co-creation, in which the shared knowledge of scientists and decision makers is particularly needed during the uncertainty of a crisis.

Decision makers also need to be able to commission research, including implementation research, between emergencies rather than only reacting after them and preparing for the last outbreak; as the WHO Hub noted, this is not easy for public health agencies, which are not science ministries or research bodies themselves. Addressing this requires more than one-off efforts: it calls for institutional arrangements that bring researchers and decision makers together routinely, help them ask the right questions, and set priorities according to policy needs.

## Institutionalising evidence use in health systems

To reinforce country capacity and make evidence use a regular part of health policy, programmes and investments, WHO established the Evidence-informed Policy Network (EVIPNet). WHO’s approach considers how evidence use becomes embedded in the structures, processes and culture of a health system.

EVIPNet pursues four connected aims: to strengthen and embed countries’ capacity to use evidence; build trust and confidence in science; adapt global evidence and WHO guidance to national priorities and circumstances, often described as “glocalisation”; and build partnerships among the many organisations that produce, interpret and use evidence [8]. National platforms can link ministries with researchers, universities, civil society and other partners so that evidence reaches decisions and is considered alongside local needs.

Its accompanying guidance and checklist examine six domains: governance and accountability; standards and routine processes; leadership and commitment; resources, including staff and technical capacity; organisational culture; and partnerships across institutions [8, 9]. Together, these domains help assess whether evidence can be brought into decisions consistently and sustained as circumstances and leadership change. At the centre is knowledge translation, the work that connects evidence with the questions decision makers need to answer.

Ethiopia offered an example of how these arrangements take shape within a country. After grappling for some time with different frameworks for assessing the country’s evidence ecosystem, the Ethiopian Public Health Institute’s knowledge translation team welcomed the WHO checklist, adapted it, and used it to see where the ecosystem stood across the six domains. Dedicated institutional mandates for knowledge translation and evidence-informed decision-making were in place, and structures such as technical working groups, research advisory councils and informal “brown bag” sessions in which researchers present findings to high-level decision makers were seen to work well in bringing evidence into policy discussions. Surveillance was fairly strong from subnational to national level. The assessment also identified gaps. Knowledge translation capacity was growing but concentrated in a few, mainly governmental, institutions. Tools and processes for presenting evidence to decision makers were not yet standardised or harmonised, which created a barrier when evidence was brought to them. Combining surveillance data with evaluation and research findings, and contextualising them for decision making, remained the main challenge.

In Kenya, the SCINOP project, which follows a county-led knowledge translation model, drew on the WHO evidence-informed policy-making checklist to develop a locally adapted science-for-policy curriculum with Moi University and county health teams. The University of the West Indies centre in Trinidad and Tobago has more recently examined how these activities can become a regular part of national decision processes. With support from WHO EVIPNet and University College London, it assessed the country’s health evidence system using the six domains of the WHO checklist described above. The assessment led to an action plan with concrete actions to strengthen the routine use of evidence during both normal operations and emergencies. Its overarching recommendation is a national Evidence to Action Council for Health to provide strategic oversight and coordination and to implement the plan. The council had not yet been established at the time of the session, and the assessment report was being finalised, so its full set of recommendations was not presented. The centre’s aim is to move from individual products and training activities towards arrangements that make evidence available to decision makers during both ordinary operations and emergencies.

WHO also noted that interaction between researchers and decision makers should be accompanied by strengthened capacity to formulate research and evidence synthesis questions. EVIPNet bases review questions on identified policy priorities, and institutions such as the EPPI-Centre at University College London have tools for commissioning evidence synthesis, while other organisations have developed tools on using and commissioning implementation research. WHO also stressed that research priorities should be set according to policy relevance and not simply because gaps in research exist, and linked to foresight exercises relevant to decision making.

Once the right evidence has been generated, it still has to be translated. That is the work of knowledge translation, which sits at the centre of the WHO framework.

## Knowledge translation in practice

Knowledge translation involves bringing together, exchanging and applying knowledge with the people who need to use it. The Johns Hopkins Bloomberg School of Public Health, in line with the WHO definition, defines it as the synthesis, exchange and application of knowledge by relevant stakeholders to accelerate the benefits of global and local innovation for health systems and people’s health [10]. It involves purposeful efforts to address barriers to evidence use, typically engages several stakeholders and both health and non-health evidence, such as economic data, and often takes the form of a knowledge translation organisation or platform. Of the models for linking research to action [11], much of the effort has gone into integrated approaches, in which entities help align the work of those generating evidence with the needs of those who use it in policy and decision making. In an outbreak, this work has to be done with less certain evidence, greater conflict over its meaning and much less time than in routine policy making, as the example of Ethiopia above shows.

At county level, SCINOP works with two counties, Bungoma and Busia, to make evidence use part of routine planning and decisions. A county-level taskforce and agreements with county leaders have helped include evidence activities in budgets and annual work plans. The project has also brought evidence into wider discussion. A maternal, newborn and child health summit convened researchers, county and national officials, and other partners to discuss local priorities and how national policies work at county level. Cross-border policy dialogues and an evidence-based documentary, a film drawing on local evidence, have raised issues for county and national discussion. The project plans to explore AI to speed the preparation of policy briefs and make local research, reports and health guidance easier to find and use. County health management teams would help shape this work around the questions they face when making timely decisions. The team also linked its county work to wider knowledge translation discussions through the AMPATH Knowledge Translation Core (AMPATH is a Moi University–led health partnership in Kenya).

To address the gaps identified in its assessment, the Ethiopian Public Health Institute is extending knowledge translation capacity to regional and sub-regional levels through training of trainers and capacity building in policy briefs, rapid reviews and the concept of knowledge translation itself. It is also working with the national health evidence network to harmonise the tools used to present evidence to decision makers, and to strengthen how surveillance data are combined with evaluation and research findings for decisions.

Outside emergencies, the Ethiopian Public Health Institute runs monitoring and evaluation of its knowledge translation work. When a policy brief has been developed, provided to decision makers and discussed in a dialogue, the Institute assesses whether it has been useful or applied in the system and, if so, which factors helped. It noted that this system can be strengthened, and that the knowledge translation process is an area where learning is still limited, which is also a focus of global initiatives. Such follow-up is costly and needs an integrated system that is still being built, and is not yet one of the Institute’s strongest features. These examples show that knowledge translation, like evidence use more broadly, depends on conditions beyond the work itself, such as political commitment, resources and institutional support, which are not always easy to sustain.

## Sustaining evidence use and knowledge translation: challenges and responses

Across the examples discussed in this report, producing and translating evidence was only one part of getting it into decisions. Evidence needed to answer a question that decision makers recognised, reach them in time, and be presented in a form they could use. A number of challenges were recognised during the session.

Political commitment and continuity were a first challenge. In Trinidad and Tobago, a change of government meant that sensitisation, advocacy and training had in effect to begin again with new decision makers. The centre has responded by working with champions within government ministries, regional health authorities and universities. Kenya faced a similar difficulty in securing political buy-in for its initiative and sustaining momentum across political cycles, with a general election due in 2027. To reduce these risks, the project used knowledge translation platforms to align evidence activities with changing political priorities, including local research committees within county governments, formal anchoring of evidence activities in county annual work plans, and routine policy dialogues that make evidence-based workflows part of regular practice. These measures aim to change the mindset so that evidence is treated as a critical component of every decision and policy-making cycle, to build consensus on the kind of evidence needed for different decisions, and to institutionalise cultural change.

Resources and funding were a second. In Trinidad and Tobago, with the rapid response service unfunded, the centre’s offer of rapid evidence briefs during COVID-19 was not taken up. Instead, CCHSRD formed a local research group within the Faculty of Medical Sciences to generate evidence relevant to COVID-19 prevention, treatment and mitigation. As attention shifted to preparedness for future emergencies, it co-produced a policy brief and held a stakeholder dialogue on the mental health of health workers in Trinidad and Tobago. The work asked what could be learned from COVID-19 and which measures could better protect health workers in a future emergency. The centre’s integrated knowledge translation approach, built on collaboration, capacity building and co-production, is one way in which it has been able to achieve some impact in a resource-constrained setting.

Institutional sustainability was a further concern. Ethiopia’s embedded infrastructure helps keep evidence use routine, even though these structures face challenges in the current global context.

Learning and accountability were a further theme. After-action reviews were discussed as one route to learning from emergencies. The Ethiopian Public Health Institute’s team contributed evidence to the country’s COVID-19 after-action review, which brought valuable lessons on the response itself, but the review gave limited attention to how evidence had moved into decisions. The moderator suggested that evidence-to-policy processes should become an explicit part of after-action reviews. WHO noted that it had not carried out after-action reviews systematically following COVID-19, although it convened a global evidence-to-policy summit in 2021 to share lessons across countries [12], and is preparing a guide and a community of practice on health systems learning. To keep the momentum gained during COVID-19, if not the urgency, the Institute is working to attach the use of evidence to accountability, although how to bring real accountability to evidence use in the health system is still being worked out.

Kenya’s project has also sought to build demand for evidence beyond government. It aims to create feedback mechanisms through greater community participation, to build a more scientifically engaged citizenry, and to strengthen cross-border health through dialogues across counties and with neighbouring countries.

Artificial intelligence was discussed as both an opportunity and a risk. The Kenyan team sees great potential for AI to change how decisions and policies are made, by improving the efficiency of data-driven decision making and bringing out how different indicators and the felt needs of communities fit together, drawing qualitative insights closer to quantitative parameters. Its thinking has focused on generative AI and large multimodal models to increase access to evidence, to give real-time prompts on where quick, impactful decisions can be made with efficient use of resources, and to strengthen evaluation and monitoring of service-level indicators. The team stressed that the effectiveness and ethical use of AI, trust, and the risk of misinformation through hallucinations need comprehensive safeguards, an area addressed by WHO guidance on the ethics and governance of large multi-modal models [13].

WHO agreed that AI can do a great deal of the work in evidence synthesis, where methods and technology are developing quickly, but considered that relationship building, brokerage and deliberative dialogue between researchers and decision makers are difficult to see being replaced. The moderator shared this concern, noting that AI could undermine the personal relationships that are central to evidence-informed policy. The moderator concluded that a forward-looking agenda is to use AI so that it strengthens, and does not undermine, the evidence-to-policy process.

## Moving forward

The session showed that timely evidence depends on work done before a question becomes urgent. Countries can establish mandates, teams and regular processes; create space for decision makers to define what they need; and connect surveillance findings with research, evaluation and local experience. These arrangements need staff, resources and relationships that can continue beyond a single project or emergency.

The examples also leave open questions. Ethiopia is working to carry the capacity built during COVID-19 into routine decisions and beyond national institutions. Trinidad and Tobago has developed an action plan and is working towards a national council to take it forward. Kenya is building evidence use into county plans and exploring AI-assisted synthesis. Future work should examine whether these approaches change decisions in practice, how they can be sustained, and how uncertainty and competing priorities are made clear to those deciding and to the public.

When asked what single change would most help decision makers receive the evidence they need, panellists gave complementary answers. Trinidad and Tobago pointed to consistent access to high-level committees and executive leadership, which would allow a rapid response service to be established. Such services are usually triggered by a question from a policy maker that, once agreed with researchers, is answered with a rapid synthesis, and established models exist, including those at the McMaster Health Forum and the Knowledge to Policy Center in Lebanon, both WHO collaborating centres [14, 15]. WHO emphasised the enabling environment: without trust in science and a political economy that allows evidence to be used, investment in systems, mechanisms and standards may be in vain. In Brazil, where evidence use in health is institutionalised, the other pillars of the framework were mature enough to maintain the system when political commitment decreased, and the process has been re-established under a new government, but growing mistrust in science destabilises the whole system. Ethiopia proposed building the capacity of citizens and users to ask for evidence to be used in action, as a route to real accountability that is already seen in election seasons. Kenya called for a move from static evidence to living evidence and user-driven synthesis, shifting the focus from research organisations to end users in day-to-day policies and decisions.

In closing, the moderator noted how far evidence-to-policy work has developed since initiatives such as EVIPNet began in the mid-2000 s. Personal relationships remain critical, but Ethiopia’s experience showed how they can be transformed into institutional relationships between organisations, so that they do not rely on personal relationships that political change can easily disrupt. Specific tools and approaches, such as reverse research days, rapid reviews and living evidence documents, offer newer ways of summarising evidence in timely, usable formats, and better alignment of tools was identified as a further need. The WHO Hub noted that researchers and policy makers share an interest in research being used, which should make closer collaboration an obvious win–win, but that structural challenges in how governments and research organisations are set up still stand in the way.

Moving forward, the experiences shared in this session point to a common conclusion: evidence systems are a foundation of health systems, not only a tool for emergencies. Building them in ordinary times, through mandates, relationships and routine processes, is what allows evidence to reach decisions when the next crisis comes.

## Data Availability

Not applicable.

## Abbreviations

AI: Artificial intelligence
AMPATH: Academic Model Providing Access to Healthcare
CCHSRD: Caribbean Centre for Health Systems Research and Development
COVID-19: Coronavirus disease 2019
EPPI-Centre: Evidence for Policy and Practice Information and Co-ordinating Centre
EVIPNet: Evidence-informed Policy Network
SCINOP: Driving Scientific and Knowledge-to-Policy Adaptation in Maternal Child Health
WHO: World Health Organization
